# Chinese Tuina Protects against Neonatal Hypoxia-Ischemia through Inhibiting the Neuroinflammatory Reaction

**DOI:** 10.1155/2020/8828826

**Published:** 2020-12-31

**Authors:** Pengyue Zhang, Qian Zhang, Bowen Zhu, Shijin Xia, Xianyan Tai, Xiantao Tai, Bing Li

**Affiliations:** ^1^Key Laboratory of Acupuncture and Tuina for Treatment of Encephalopathy, College of Acupuncture, Tuina and Rehabilitation, Yunnan University of Traditional Chinese Medicine, Kunming 650500, China; ^2^Zhejiang Provincial Hospital of TCM, Zhejiang Hangzhou 310006, China; ^3^Department of Geriatrics, Shanghai Institute of Geriatrics, Huadong Hospital, Fudan University, Shanghai 200040, China; ^4^Department of Prevention and Health Care, The First Hospital Affiliated to Kunming Medical University, Kunming 650032, China; ^5^Jinshan Hospital of Fudan University, Shanghai 200054, China

## Abstract

*Aim*. Neonatal hypoxic-ischemic encephalopathy (HIE) is a significant cause of perinatal morbidity and mortality. Chinese Tuina is an effective treatment for HIE, but the molecular mechanisms are yet unknown. This study investigated the effect and mechanisms of Chinese Tuina on the inflammatory response in neonatal HIE rats. *Main Methods*. 30 male neonatal rats were divided randomly into 3 groups: sham, HIE, and HIE with Chinese Tuina (CHT) groups. The HIE and CHT groups were subjected to left common carotid occlusion and hypoxia at 3 days postnatal (P3). The pups in the CHT group received Chinese Tuina treatment on the next day for 28 days. The weight was measured at P4, P9, P13, P21, and P31. The behavioral functions were determined at P21. The protein expression and the methylation level in promoter regions of TNF-*α* and IL-10 were determined by Western blotting, immunohistochemistry, and pyrosequencing, respectively, at P33. *Key Findings*. The weight gain in the HIE group was slow compared with that of the CHT group. The rats in the CHT group performed better both in the balance beam and hang plate experiment. Chinese Tuina inhibited the expression of TNF-*α* and upregulated the expression of IL-10. Neonatal hypoxic-ischemic injury downregulated the methylation level in promoter regions of TNF-*α* at all CpG points but not IL-10. However, Chinese Tuina did not change the methylation level in promoter regions of TNF-*α* and IL-10. *Significance*. Chinese Tuina protected against HIE through inhibiting the neuroinflammatory reaction. While HIE markedly downregulated the methylation level of TNF-*α*, the protective effects of Chinese Tuina were independent of the regulation of the methylation level of TNF-*α* and IL-10.

## 1. Introduction

Neonatal hypoxic-ischemic encephalopathy (HIE) is a significant cause of perinatal morbidity and mortality. HIE damages the gray matter, including the sensorimotor cortex, basal ganglia, and thalamus, and results in abnormal white matter development [1, 2]. This leads to severe neurological deficits, such as cerebral palsy, mental retardation, seizures, learning disabilities, and other neurological disorders [3–5]. Although there is an increased understanding of the mechanisms underlying the pathophysiology of HIE, there are no current well-established treatments for HIE [6, 7]. Thus, it is essential to further study the pathophysiology of HIE and discover new treatment strategies and effective interventions.

Massage has significant effects on child development, especially for motor function disorders of children affected by cerebral palsy. The more early the treatment, the more effective is the massage therapy [8, 9]. Massage can increase the distribution of blood and warmth to distal regions by pressing the major signal point [10, 11]. Chinese Tuina is a branch of massage technology that is widely used for spinal joint diseases or visceral diseases and involves manipulating spinal tissue, bone, joint, and meridians [12]. In Western countries, the corresponding treatment technology is called chiropractic or spinal manipulation, which refers to the dysfunction caused by spinal and adjacent subluxation, and is treated with specific treatment techniques [13]. These techniques can relieve skeletal muscle pain, shorten the recovery time of pain, improve the range of activity, correct the pathological deformity, and improve the physiological response in the central nervous system [14, 15]. Our previous clinical trial found that a three-month Chinese Tuina treatment alleviated muscle cramps and improved joint activity in cerebral palsy-affected children [16]. Furthermore, our animal experiments showed that Chinese Tuina therapy promoted growth and movement function recovery of hypoxia-ischemia injured SD rats [17]. However, the underlying molecular mechanism responsible for the neuroprotection of Chinese Tuina remains poorly understood.

The neuroinflammatory reaction is considered to be a critical pathophysiological factor in the development of an immature brain. The initial inflammatory response to hypoxic-ischemic injury results in secondary neuronal injury [18, 19]. Hypoxia-ischemia initially activates microglia, the resident immune cells in the brain, and then disrupts the blood-brain barrier (BBB), leading to peripheral leukocyte infiltration, which further intensifies inflammation and brain damage [20, 21]. Thereafter, a multitude of inflammatory cytokines are secreted by immune cells and activated astrocytes, which induces neuronal apoptosis, inhibits neurogenesis, and hampers repair of the brain [22]. Our previous research provided evidence that expression of proinflammatory cytokines including TNF-*α* was upregulated and expression of anti-inflammatory cytokines such as IL-10 was downregulated in patients [23, 24]. Evidence from neonatal animals demonstrated that inhibition of the inflammatory reaction is beneficial to recovery from the hypoxic-ischemic insult [25–27]. Thus, the neuroinflammatory reaction is a key therapeutic target for the treatment of neonatal ischemic brain damage.

DNA methylation is an epigenetic mechanism connected with somatopsychic fitness and disease [28]. It is also a crucial cellular mechanism for regulating gene expression associated with inflammatory processes [29]. DNA methylation can be modified and subsequently influence behavior and gene expression through early life experiences, as shown by studies in humans and animals [30–32]. In this study, we used an experimental neonatal hypoxic-ischemic rat model to investigate the effects of Chinese Tuina on the neuroinflammatory reaction, as well as the underlying mechanism.

## 2. Materials and Methods

### 2.1. Animals and Grouping

Animal experiments were carried out according to the regulations of Shanghai health clinical center laboratory animal welfare and ethics committee: N0.2019-A018-02 in Shanghai, China. Pregnant Sprague-Dawley (SD) rats (gestation age: 14-18 d) weighing 340-380 g were purchased from Shanghai Slac Laboratory Animal Co. Ltd. Before natural birth, the rats were reared at 24 ± 2°C with a 12-hour day and night cycle, in the animal laboratory of the Jinshan Hospital, which is affiliated with Fudan University, Shanghai. Relative conditions of humidity were 40%~70%. Water and food intake was available ad libitum. To avoid fighting, pregnant rats were housed separately. A total of 30 male pups were included in this study, which were randomly assigned to the hypoxic-ischemic model. The groups were the Chinese Tuina group (CHT group), sham control group (sham group), and hypoxic-ischemic model group (HIE group) with 10 rats in each group. The cortex tissue of 5 rats was used for WB and methylation detection experiment; limited by fund, the rats in the methylation detection experiment were 3. And 5 rats were used for the immunohistochemistry experiment.

### 2.2. Hypoxic-Ischemic Model and Testing

Three-day-old rats were used to prepare the hypoxic-ischemic model after their natural delivery by pregnant rats, as previously described [33]. The rats lying on their back on the operating table were anesthetized with isoflurane, and then, the neck skin was opened under a dissection microscope. After separating and ligating the left carotid artery, the wound was sutured, and then, they were placed in an airtight hypoxic box for 2 hours at 37°C temperature, 8% oxygen, and 92% nitrogen gas mixture.

The righting reflex experiment was used to detect whether the model was successful or not. The experiment was performed before weighing at P4 (4 days postnatal). The operation was initially to lift the tail of the rat pup and place its back on a horizontal board at first. Then, the operator placed their right index finger against the mandible of the rat pup and the right thumb on the hind legs. The two fingers of the right hand were then released while the left hand used a stopwatch to start the time. Each of the rat pups was tested twice, and rat pups that could not return to the normal position from an abnormal position in 20 seconds were placed into the hypoxic-ischemic group of pups.

### 2.3. Manipulation Method

A professional, certified physician was responsible for the Chinese Tuina intervention. On the first day after the HIE operation, the rats in the CHT group were given the Chinese Tuina intervention for 28 days with a frequency of Chinese Tuina manipulations of 120 per minute, which continued for 15 minutes. While the CHT group carried out the Chinese Tuina intervention, the sham group and the HIE group of pups were kept in their cages with no intervention. The Chinese Tuina intervention consisted of putting the pups with the face down on a slightly bent palm of the left hand, which provided balance and allowed pups to adapt to the environment. Thereafter, the middle finger of the right hand was used to do gentle Chinese Tuina from the back of the neck of the pup to the tail two or three times to relax the muscles. The dorsal and bilateral ligaments and muscles (called the Du meridian and bladder meridian, in traditional Chinese medicine (TCM)) of the pups were subjected to rubbing manipulation and pushing manipulation with one finger and a kneading manipulation from top to bottom, center to side [17].

### 2.4. Weight

To observe the growth and development of young rats, their weights were measured at P4, P9, P13, P21, and P31. The behavioral assessment including weight, balance beam experiment, and tilting plate experiment was performed by a laboratory assistant blinded to experiment design.

### 2.5. Balance Beam Experiment

The balance during movement was measured using a balance beam (35 cm long, 1.5 cm wide, and 100 cm high). Each rat starting from P21 was trained once a day for 3 consecutive days, and the height was gradually increased in preparation for the balance beam test. The test was conducted three times, and the mean of measurements was determined. Dates were recorded, as was the time of the initial setting, the time when the rat first entered the dark box (safety area), and the number of times when the rat's hind legs slipped on the balance beam.

### 2.6. Tilting Plate Experiment

The experiment was based on Rivlin's method to carry out a tilting plate experiment, which measured the recovery of motion function at P21 [34]. We placed the rat head forward, and the body longitudinal axis paralleled to the longitudinal axis of the inclined plate. The angle of the plate was increased gradually from small to large, until the maximum angle was achieved where the rat stayed on the plate for 5 s without dropping. This maximum angle was the behavior score of the slanting experiment. Each rat was tested 3 times, and the results were averaged.

### 2.7. Western Blot

These rats from each group were euthanized on the 33rd day after birth with 5% isoflurane. And the brains were obtained; the total proteins from the cerebral cortex of rats (*n* = 5) were extracted, and the protein concentration was measured by a BCA kit (Thermo). Then, the total protein was separated on 10% SDS-PAGE gels (Beyotime Biotechnology). Proteins on SDS-PAGE were transferred to a PVDF membrane, which was immersed in TBST blocking solution containing 5% skim milk powder and blocked at room temperature for 2 h. After removing the blocking solution, the PVDF membrane was cut according to the prestained protein standards and incubated in primary antibody against TNF-*α* and IL-10 rabbit polyclonal antibodies (Abcam) for 24 h at 4°C. An internal reference protein (*β*-actin) was applied in this study. After that, the membranes were washed three times by TBST (PBS containing 0.1% tween-20) and incubated in HRP-conjugated anti-rabbit IgG (Jackson ImmunoResearch Laboratories) for 1 h at room temperature. After three washes, the protein signals were determined by the gel imager and image analysis system (Tanon Technology Co., Ltd., Shanghai).

### 2.8. Immunohistochemistry Staining

Rat pups were deeply anesthetized with 5% isoflurane on the 33rd day after birth and transcardially perfused with physiological saline and 4% paraformaldehyde. The brains were gained and dehydrated in 20% sucrose solution overnight. Then, these brains were cut into slices of 30 *μ*m thick on the freezing microtome (SLEE, Germany). These slices were permeabilized with 0.2% Triton X-100 for 15 min and were then blocked with 10% normal goat serum for 1 h at room temperature. After that, they were incubated with primary antibody against TNF-*α* and IL-10 rabbit polyclonal antibodies (Abcam) for 1 hour at room temperature and then at 4°C overnight. On the next day, they were washed with PBS and incubated with biotinylated goat anti-rabbit IgG for one hour at room temperature. And then, they were treated in an avidin-horseradish peroxidase complex (Vectastain Elite ABC-Reagent, Vector) for 30 min and colored with diaminobenzidine (Sigma-Aldrich). The positive signals were digitized with a microscope (Nikon, Japan).

### 2.9. The Detection of Level in Methylation by Pyrosequencing

Genomic DNA extraction kits (Qiagen Co., Ltd., No. 51306) and methylation modification kits (Qiagen Co., Ltd., No. 59104) were used. The DNA methylation analysis by Sequenom mass spectra was carried out by Shanghai Benegene Biotechnology Co., Ltd. The gene sequence information of IL-10 and TNF-*α* in rats was obtained through the NCBI (National Center of Biotechnology Information) website. Primers for these genes were designed by PyroMark Assay Design 2.0 and synthesized by BGI Genomics (Tables 1 and 2). Rats (*n* = 3) were sacrificed to extract DNA with a genomic DNA extraction kit (Qiagen Co., Ltd., No. 51306) on the 33rd day. The DNA was modified with a bisulfate reagent with a methylation modification kit (Qiagen Co., Ltd, No. 59104). PCR amplification of the pyrosequencing template was performed using primers in Table 2. PCR amplification conditions were 95°C for 3 min, 94°C for 30 s, 56°C for 30 s, and 72°C for 1 min, 40 cycles. Pyrosequencing and the frequency of methylation were determined by PyroMark Q96 ID (Qiagen) [35]. Pyro Q-CpG software automatically analyzed the methylation state of each site.

F: upstream primer; R: downstream primers; S: sequencing primers; biotin: labeled biotin.

### 2.10. Statistical Analysis

The data were presented as means ± standard deviation (SD). The test standard was *α* = 0.05. Single factor analysis of variance (one-way ANOVA) was used to compare the differences among groups of measurement data. The LSD *t*-test (Fisher's least significant difference *t*-test) was used for pairwise comparison of multiple measurement data. A value of *P* < 0.05 was considered to be statistically significant.

## 3. Results

### 3.1. Chinese Tuina Promoted the Growth and Development in Neonatal Hypoxic-Ischemic Rats

**Figure 1:**
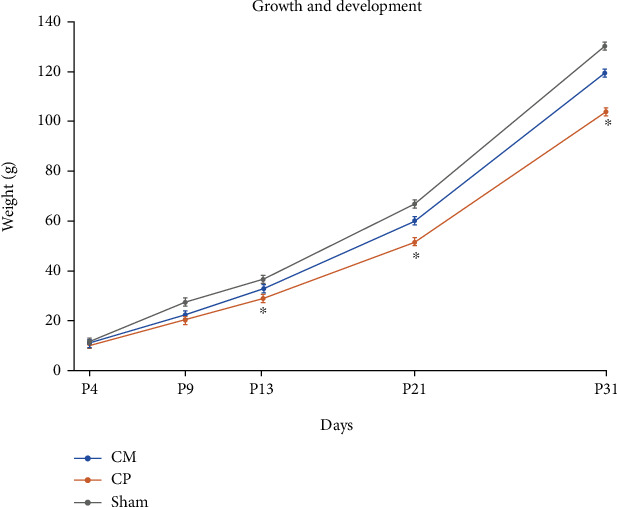
Chinese Tuina promoted growth and development in rats with HIE. ^∗^*P* < 0.05, comparison with the HIE group, *n* = 10.

The results from weight gain showed that the hypoxic-ischemic model led to delayed growth and development. Chinese Tuina promoted the growth and development in rats with HIE at postnatal days 13, 21, and 31 (the days 9, 17, and 27 of operation) compared with the CHT group (*P* < 0.05, Figure 1). Notably, there was no difference in the weight gain between the CHT group and the sham group (*P* > 0.05 for all test points). These results indicated that rats in the HIE group were slow to gain and grow, whereas Chinese Tuina promoted the growth and development in neonatal hypoxic-ischemic rats.

### 3.2. Chinese Tuina Improved Behavioral Recovery in Neonatal Hypoxic-Ischemic Rats

**Figure 2:**
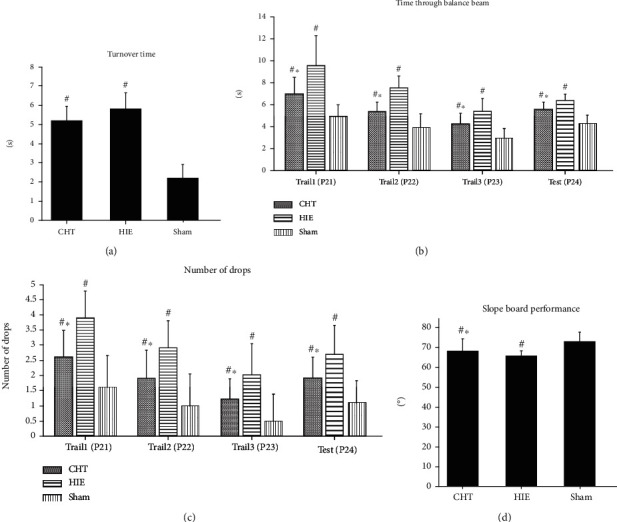
Chinese Tuina improved behavioral recovery in rats with HIE. ^∗^*P* < 0.05, comparison with the HIE group, ^#^*P* < 0.05: comparison with the sham group, *n* = 10.

The hypoxic-ischemic treatment led to significant impairment in righting reflex in both the CHT and HIE groups compared with the sham group on day 4 postnatal (*P* < 0.05, Figure 2), whereas there were no statistical differences between the CHT group and the HIE group (*P* = 0.293 > 0.05). This result showed that the hypoxic-ischemic model was successful. The motor and balance function recovery in the balance beam test showed that Chinese Tuina improved the time taken to pass through the balance beam and reduced the number of drops from the balance beam (*P* < 0.05, Figures 2(b) and 2(c)). However, these beneficial effects induced by Chinese Tuina did not reach the level of the sham group (*P* < 0.05, compared with the sham group, Figures 2(b) and 2(c)). Similarly, the rats in the CHT group had a steeper slope than rats in the HIE group (*P* < 0.05, Figure 2(d)), while the rats in the sham group had the steepest slope among the three groups. These results indicated that Chinese Tuina promoted the motor and balance function recovery in neonatal hypoxic-ischemic, rats but the recovery was not complete.

### 3.3. Chinese Tuina Improved the Neuroinflammatory Reaction in Neonatal Hypoxic-Ischemic Rats

**Figure 3:**
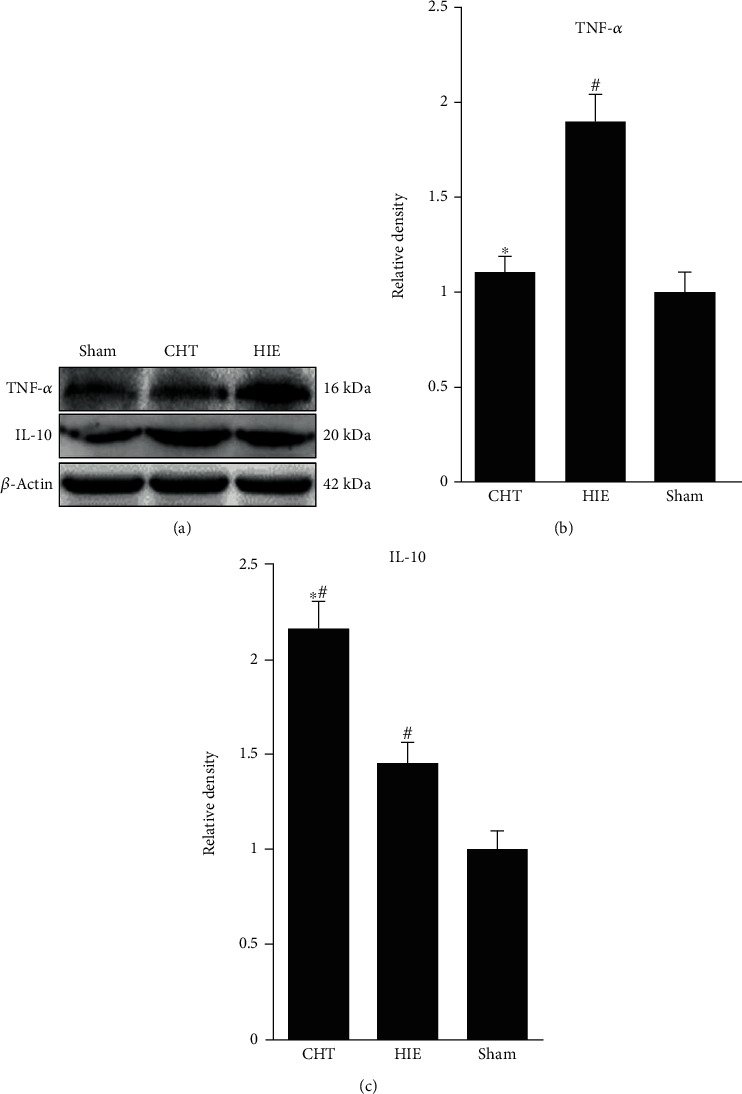
Chinese Tuina improved the neuroinflammatory reaction in rats with HIE. (a) Representative images of Western blotting for TNF-*α* and IL-10. (b, c) Quantification of the optical density for TNF-*α* and IL-10, normalized to *β*-actin. ^∗^*P* < 0.05, comparison with the HIE group; ^#^*P* < 0.05, comparison with the sham group, *n* = 5.

TNF-*α* is a key proinflammatory cytokine in neonatal hypoxic-ischemic encephalopathy and prevents nerve growth and repair. Western blot analysis showed that 4 weeks of Chinese Tuina inhibited the expression of TNF-*α*, which attained the level of the sham group (Figures 3(a) and 3(b)).

IL-10 has an important anti-inflammatory action, and its upregulated expression is beneficial to the neuroplastic niche in brain disease. We measured the expression of IL-10 by Western blot, and the results showed that neonatal hypoxia-ischemia upregulated the expression of IL-10 compared to the sham group and that Chinese Tuina increased the expression of IL-10 compared to the HIE group.

These findings suggest that Chinese Tuina improves the neuroplastic niche in neonatal hypoxic-ischemic rats through the decreased expression of proinflammatory cytokine TNF-*α* and increased expression of anti-inflammatory cytokine IL-10.

### 3.4. Evaluation of Neuroinflammatory Reaction by Immunohistochemistry

**Figure 4:**
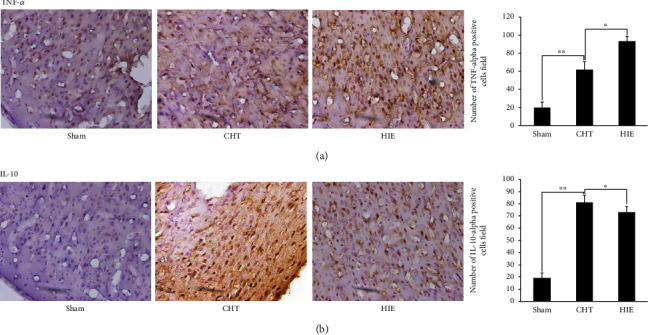
Chinese Tuina improved the neuroinflammatory reaction in rats with HIE. (a) Representative photomicrographs of TNF-*α*-positive cells (40x) and the quantitative results of the number of positive cells; (b) representative photomicrographs of IL-10-positive cells (40x) and the quantitative results of the number of positive cells.

To further investigate the effects of Chinese Tuina on the neuroinflammatory reaction, we detected the expressions of TNF-*α* and IL-10 by immunohistochemistry. Similar to the results of Western blot, Chinese Tuina significantly decreased the positive cells of TNF-*α* and increased the positive cells of IL-10 in the cortex of neonatal hypoxic-ischemic rats (Figures 4(a) and 4(b)).

### 3.5. The Effects of Chinese Tuina on the Methylation Level of Cytokines TNF-*α* and IL-10 in Neonatal Hypoxic-Ischemic Rats

**Figure 5:**
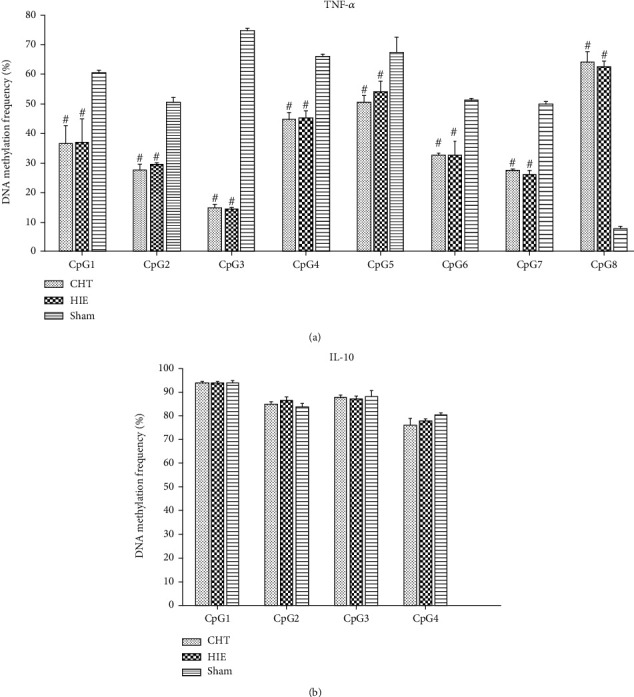
DNA methylation level in the promoter region of cytokines TNF-*α* and IL-10. ^#^*P* < 0.05: comparison with the sham group, *n* = 3.

Through sequence analysis, we chose 8 and 4 CpG islands in the promoter regions of the TNF-*α* and the IL-10 gene, respectively. While the results showed that the hypoxic-ischemic injury markedly changed the methylation status in the promoter region of TNF-*α* at all of the tested CpG islands, the Chinese Tuina intervention did not change the methylation status in the promoter region of TNF-*α* compared to the HIE group (Figure 5(a)). Interestingly, the neonatal hypoxic-ischemic model led to reduced methylation levels at CpG islands 1-7 in the TNF-*α* promoter region but increased the methylation level at the 8^th^ CpG island. The results indicated that the neonatal hypoxic-ischemic model resulted in changes in the methylation status in the promoter region of TNF-*α*. However, the neonatal hypoxic-ischemic model did not change the methylation status in the promoter region of IL-10, and Chinese Tuina did not change the methylation status in the promoter region of IL-10 compared to the HIE group (Figure 5(b)).

## 4. Discussion

The neuroinflammatory reaction is the major detrimental factor within the neuroplastic niche in nervous system disease. The improved neuroinflammatory reaction is critical for renewal and repair. In the present study, we found that neonatal hypoxia-ischemia markedly downregulated the methylation level of TNF-*α* but did not change the methylation level of IL-10. Chinese Tuina may improve the neuroplastic niche in neonatal hypoxic-ischemic rats through inhibition of the expression of the proinflammatory cytokine TNF-*α* and increasing the expression of the anti-inflammatory cytokine IL-10. However, the beneficial effects of Chinese Tuina were independent of the regulation of methylation levels in the TNF-*α* and IL-10 promoter regions.

Chinese Tuina, massage, and touch therapy was widely applied to the prevention and treatment of pediatric diseases. Touch therapy promoted the maturation of the autonomic system, heart rate, and respiration and circadian systems through skin-to-skin contact between a mother or physiotherapists and an infant [36]. And massage enhanced vagal activity and reduced cortisol levels through the stimulation of pressure receptors [37]. Chinese Tuina regulated the circulation of Qi and blood, unblocked the impaired meridians, and promoted the recovery of function. The therapy method of Chinese Tuina was a series of orderly manual techniques on the surface of the body. These manual techniques included press, knead, knock, and friction. Although Chinese Tuina, massage, and touch therapy belonged to noninvasive therapy through using various manual techniques, the choice of locations on the body and the specified manual techniques of Chinese Tuina were based on Traditional Chinese Medicine (TCM) zang-fu organs and meridian theory. Chinese Tuina paid more attention to unblocking meridians and the balance of Yin and Yang [38]. Previous studies showed that Chinese Tuina improved learning ability and memory in rat pups with hypoxic ischemia. The mechanism may involve the regulation of monoamine neurotransmitters such as 5-HT, norepinephrine (NE), adrenaline (E), and dopamine (DA) in the hippocampus [39].

The cerebral cortex and hippocampus show obvious abnormal inflammatory responses and are involved in the development of neonatal hypoxic-ischemic injury [40, 41]. The most important neuropathological changes in the brains of children with hypoxic ischemia are in the area of cerebral white matter injury accompanied by periventricular leukomalacia (PVL) [42]. This injury is due to the high sensitivity of some nerve cells to ischemia and hypoxia. Chronic cerebral ischemia and hypoxia increase the release of proinflammatory cytokines and promote the apoptosis of oligodendrocytes, which are the main cells that make up the white matter of the brain [41, 43, 44]. Therefore, we suspect that abnormal regulation of neurotransmitters is closely related to the occurrence and progression of hypoxic ischemia, and this is mediated by inflammatory factors. Variations in DNA methylation are an important inflammatory regulatory mechanism in hypoxia-ischemia [45, 46]. Our results confirmed that neonatal hypoxia-ischemia markedly decreased the methylation level at CpG islands 1-7 in the TNF-*α* promoter region, which is a critical proinflammatory factor in neonatal hypoxic-ischemic encephalopathy. Although the methylation position at the 8^th^ CpG island was increased in the CHT group, the methylation level in the whole methylated region was decreased. Thus, the transcription and the protein expression of the TNF-*α* were increased. This result had been confirmed by Western blot detection which showed that the protein expression of TNF-*α* was significantly upregulated in the HIE group compared with the sham group. However, Chinese Tuina inhibited the upregulation of TNF-*α* which was detected by Western blot. The possible reason was posttranscriptional regulation. The undergoing mechanisms need further study.

In this study, to compensate for the known effect of estrogen [47], we only included male rat pups in the study and we carried out the righting reflex experiment to substantiate the hypoxic-ischemic model [48]. Further support for the model was obtained at the behavior and molecular levels. This study has several novel findings: (i) Chinese Tuina promoted weight gain of hypoxic-ischemic rats, shortened the time taken to pass through the balance beam, and increased the angle at which the rat pups stayed on the inclined plate. These data suggested that Chinese Tuina was beneficial to the development of motor and balance function in the hypoxic-ischemic rats. (ii) The cerebral cortex of hypoxic-ischemic rat pups had an inflammatory response, which involved the pro- and anti-inflammatory factors TNF-*α* and IL-10, respectively. Chinese Tuina improved the neural inflammatory response through reduced protein expression of TNF-*α* and increased protein expression of IL-10. These results suggest that Chinese Tuina therapy may confer neuroprotection by restoring the equilibrium in the inflammatory response niche in neonatal hypoxic-ischemic rats. (iii) The results indicated that the hypoxic-ischemic model changed the methylation status in the promoter region of TNF-*α*. However, the hypoxic-ischemic model did not change the methylation status in the promoter region of IL-10, and Chinese Tuina did not change the methylation status in the promoter region of IL-10 and TNF-*α* compared to the HIE group.

Methylation is an important epigenetic modification that regulates the expression of many genes, including those that encode inflammatory cytokines [49]. In the present study, we found that neonatal hypoxia-ischemia changed the methylation level in the promoter regions of inflammation-related genes. Thus, the regulation of methylation levels is a potential therapeutic target for the treatment of neonatal hypoxic-ischemic encephalopathy. However, the mechanism by which Chinese Tuina improved the inflammatory response in neonatal hypoxic-ischemic encephalopathy was independent of the regulation of methylation levels in IL-10 and TNF-*α*.

## 5. Conclusion

In this study, we found that neonatal hypoxic-ischemic injury markedly downregulated the methylation level in the promoter regions of the TNF-*α* gene and increased its protein expression. Meanwhile, neonatal hypoxic-ischemic injury upregulated the expression of IL-10 but did not change the methylation level in the promoter regions of IL-10. We concluded that Chinese Tuina was beneficial for improving the growth and the ability to balance in rats with neonatal HIE. The possible mechanism could be that Chinese Tuina inhibited the neuroinflammatory reaction and improved the neuroplastic niche in neonatal hypoxic-ischemic rats. But the protective effects of Chinese Tuina were independent of the regulation of methylation levels of TNF-*α* and IL-10. The undergoing mechanism needs further study.

## Figures and Tables

**Table 1:** The gene sequence information of IL-10 and TNF-*α*.

Gene	Sequence and CpG loci information
TNF-*α*	TCCCAGAGCCCATACACACTCCCAGCTCTCAAGCCACTCTGCCTTCAGCCACTTCCTCTAAGAACTCAGACAAGGGGGCTTTCCCTCCTCAA**CG**TTGAGTCTCCCCCCTTATGCAGCCAGCTGTCAGAAGCACTCCCAATGCTAAGTTCTCCCCCATGGAAGTCCTGTTTAGAAATCAAAGGGGAA**CG**GACATAGGCATGGTGTCTCTGCAGAGAAACAATGGAAGACAGAGAGGAAATGGGTTTCAGTTCCCAGGGTTCTATACATCACACACACACACACACACACACACACACACACACACACACACA**CG**TTCCTGATTGGCCC**CG**GATTGCCACAGAATCCTGGTGAGGA**CG**GAGAGGAGATTCCTTGATGCCTGGGTGTCCCCAACTTTCCAAACCTCTGCCCC**CG**CATTGGAGAAGAAACTGAGAGAGGTGCAGGGCCACTAC**CG**CTTCCTCCACATGAGATCATGGTTTTCTCCACCAAGGAAGTTTTC**CGCG**GGTTGAATGAGAGCTTTTCCC**CG**CCCTCTTCCCCAAGGGCTATAAAGGCAGC**CG**GTACACAGCCAGC**CG**GCAGAACTCAG**CG**AGGACACCAAGGGACCAGCCAGGAGGGAGAACAGCAACTCCAGAACACCCTGGTACTAACTCCCAGAAAAGCAAGCAACCAGCCAGGCAGGTTC**CG**TCCCTCTCATACACTGGCC**CG****(CpG1)**AGGCAACACATCTCCCTC**CG****(CpG2)**GAAAGGACACCATGAGCA**CG**GAAAGCATGATCCGAGATGTGGAACTGGCAGAGGAGG**CG****(CpG3)**CTCCCCAAAAAGATGGGGGGCCTCCAGAACTCCAGG**CG****(CpG4)**GTGTCTGTGCCTCAGCCTCTTCTCATTCCTGCT**CG**TGG**CG(****CpG5)**GGGGCCACCA**CG****(CpG6)**CTCTTCTGTCTACTGAACTT**CG**GGGTGAT**CG****(CpG7)**GTCCCAACAAGGAGGAGGTGAGTGCCTGGGCAG**CG**TTTATTCTCACTCACAAGCAAAGTAGGGTGGGAGGGCAAGAAAGGCAGGAGGGTAAGGGAAAGAGGT**CG****(CpG8)**GCTGGTGGGCACAGTAATGTGGAGGAGAGTTGGGGAGGGAGACCT**CG**GACC**CG**TGCAGCCAGTTGTCTAAACATCCTTGGGTGGATGCA**CG**GAGGGG**CG**AATGAA**CG**AACAAG**CG**TG**CG**TACA**CG**TGCAGAGATGTGCAGAGA**CG**TGGCCAGGGGAACAGGGAAGAAGCAAGAGATAAAAATT**CG**AGA**CG**GAGATGGGAGA**CG**AGGGAGATAAGGAGATATGAGAGATAAGGAGGGAGATGGAGGGGAAACAACAGATCAAGC

IL-10	AAGACCAAATAAGCTGAAGTTCCTGGTCCAC**CG****(CpG1)**GCTCCAGTATGGTAACCCTCTCCAATGGGACAGGCTTGGAACCCTGTACCAAAAGAGATTCTCCTC**CG****(CpG2)**TGCTGATGTGGGAAGGAGAACCCTGGGGTGTGCTCTCCACATGGGTCAACTTTTATTTAAGCAAACAGTCCCTGGCCAACAGGACATGTAGTATTGCCCTGCTTGGGTCACACAGAAAACAGGTACCAGGAGGACAAGTGGCTTGCCCAGAGCACAGAGGGAAAAGCAGTGGAGGACTCTAGGTGAATGTTCTCCCCATCCAAACTAGAAGTAGGAGAAGTCC**CG****(CpG3)**GTTGAAGGGAAGAACTAGCAGAGACTTCTGAGGACTCTGTAGAAATAATGACATCATCTCCATCCCTCCATCCTTCAACAGCCACAGGTCACACTTCTCCAAGCCTGGGA**CG****(CpG4)**TTATAAAA**CG**GGGCCATGGTGAGGACTACCTAACAGCACAGAGCAAGCTTCCTGGAAGTCTGAGCTCCTTCTCCTAACTTTCCA**CG**CTGGAATCTGAGAACCTTTACAAAACACAGGCCAGAGAAGGCACCAGAACCCTCCTCTGAT**CG**TCTGTCACACAGCCAACAAACCTTTCAAGGAAGAGTCTTGAACACACAATGGAAGAATCAAAGAGAGTGAGTTTTGAGGGTAATCAGCCCTCTCCTGTTTCCTTTGGGTAACTGAGTGCTAAGGTGACCTCCTGGTCAGCAAGAAATAG**CG**GACATTCAACCCAGGTTGAGTGGAGGAAATAATTATTTCTCAATCCTAATGTGCTCTGGAATAGCCCATTTATGCA**CG**TCATTGTGACTTA**CG**AGTG**CG**TGAATGGAACCCACAGTTGTAGATTCTCTGTACATAGAACAGCTGTCTGCCTCAGGAAATACAACTTTTAGTATTGAGAAGCTAAAAAG

**Table 2:** PCR primers and sequencing primers for CpG loci in the promoter region.

Primer	Base sequence (5′ to 3′)	Primer	Base sequence (5′ to 3′)
IL-10-1	F: AGATTAAATAAGTTGAAGTTTTTGGTTTA	TNF-*α*3	F: GAGATGTGGAATTGGTAGAGG
	R: 5′biotin-CCAAAATTCTCCTTCCCACATCAA		R: 5′biotin-ATTCTAAAAACCCCCCATCTT
	S: AGTTGAAGTTTTTGGTTTAT		S: GGAATTGGTAGAGGAG

IL-10-2	F: GGGATAGGTTTGGAATTTTGTATTAAAAGA	TNF-*α*4	F: GAGATGTGGAATTGGTAGAGG
	R: 5′biotin-CCAAAATTCTCCTTCCCACATC		R: 5′biotin-ACACTCACCTCCTCCTTAT
	S: GGAATTTTGTATTAAAAGAGATTT		S: GGGGGGTTTTTAGAAT

IL-10-3	F: TAGTGGAGGATTTTAGGTGAATGT	TNF-*α*5	F: AGAATTTTAGGAGGTGTTTGTGTTTTAGT
	R: 5′biotin-TTCTACAAAATCCTCAAAAATCTCTAC		R: 5′biotin-CCTCCCACCCTACTTTACTTATAAAT
	S: ATTAGAAGTAGGAGAAGTT		S: ATTAAGTTTTTTTGTTTATTGAAT

IL-10-4	F: AGATTTTTGAGGATTTTGTAGAAATAATG	TNF-*α*6	F: AGAATTTTAGGAGGTGTTTGTGTTTTAGTT
	R: 5′biotin-AACTCAAACTTCCAAAAAACTTACTCTAT		R: 5′biotin-ACCCTCCCACCCTACTTTACTTATAAAT
	S: TCCTCACCATAACCC		S: GTGAGTGTTTGGGTAG

TNF-*α*1	F: GGGATTAGTTAGGAGGGAGAATAGTA	TNF-*α*7	F: GGTAGGAGGGTAAGGGAAAGA
	R: 5′biotin-TAAAATTCTAAAAACCCCCCATCTT		R: 5′biotin-ATCCCAAATCTCCCTCCCCAACTC
	S: AGTAATTAGTTAGGTAGGTT		S: GGTAAGGGAAAGAGGT

TNF-*α*2	F: GGGATTAGTTAGGAGGGAGAATAGTA	TNF-*α*8	F: ATAGTAATGTGGAGGAGAGTTGG
	R: 5′biotin-CTAAAATTCTAAAAACCCCCCATC		R: 5′biotin-ACCCAAAAATATTTAAACAACTAACTACA
	S: ATTTTTTTTAGGAAAGGATATTAT		S: TTGGGGAGGGAGATT

## Data Availability

The data used to support the findings of this study are included within the article.
